# Meta‐analysis of the association of extraintestinal manifestations with the development of pouchitis in patients with ulcerative colitis

**DOI:** 10.1002/bjs5.50149

**Published:** 2019-03-13

**Authors:** K. Hata, S. Okada, T. Shinagawa, T. Toshiaki, K. Kawai, H. Nozawa

**Affiliations:** ^1^ Department of Surgical Oncology The University of Tokyo 7‐3‐1 Hongo, Bunkyo‐ku Tokyo, 113‐8655 Japan

## Abstract

**Background:**

The presence of extraintestinal manifestations may be associated with the development of pouchitis in patients with ulcerative colitis after ileal pouch–anal anastomosis. The aim of this study was to assess this correlation.

**Methods:**

A systematic literature search was performed using MEDLINE and the Cochrane Library. Studies published in English up to 22 May 2017 investigating the association between extraintestinal manifestations and development of pouchitis in adults with ulcerative colitis were included. Case reports were excluded. The association of extraintestinal manifestations with the development of overall and chronic pouchitis was investigated using a random‐effects model.

**Results:**

Of 1010 citations identified, 22 observational studies comprising 5128 patients were selected for analysis. The presence of extraintestinal manifestations was significantly associated with both chronic pouchitis (odds ratio 2·28, 95 per cent c.i. 1·57 to 3·32; *P* = 0·001) and overall pouchitis (odds ratio 1·96, 1·49 to 2·57; *P* < 0·001).

**Conclusion:**

The presence of extraintestinal manifestations is associated with development of pouchitis after ileal pouch–anal anastomosis.

## Introduction

Several treatment options are currently available for patients with ulcerative colitis^1^, ^2^. Many patients still require surgical resection for intractable disease or the development of colorectal cancer^3^. The standard surgical treatment for ulcerative colitis is restorative proctocolectomy and ileal pouch–anal anastomosis (IPAA). Anal function after surgery is generally satisfactory^4^. Minimally invasive surgery using a laparoscopic or robotic technique is also an option for IPAA^5^, ^6^. Pouchitis is a common complication after IPAA, developing in 10–58 per cent of patients according to previous reports^7^, ^8^.

Pouchitis is an idiopathic inflammation of the ileal pouch. First‐line treatment for pouchitis is antibiotics such as metronidazole and ciprofloxacin^9^. One‐third to one‐half of patients with pouchitis, however, experience chronic pouchitis. There are no evidence‐based treatments for chronic refractory pouchitis^10^, and treatments used for ulcerative colitis have been used empirically to treat chronic pouchitis. Consequently, in extreme cases of intractable chronic pouchitis, diversion or resection of the ileal pouch may be necessary.

Several risk factors for pouchitis have been reported in small retrospective cohorts; they include the presence of extraintestinal manifestations (EIMs)^11^, ^12^, primary sclerosing cholangitis (PSC)^13^, serological markers and smoking habit^14^. In a meta‐analysis^15^, the association between smoking habit and pouchitis was not confirmed. The presence of perinuclear antineutrophil cytoplasmic antibodies is a risk factor for the development of chronic pouchitis^16^. The present authors have reported previously^12^, ^17^ that the presence of EIMs was an independent risk factor for the development of pouchitis. Although some articles^11^, ^13^ have shown a similar association, this association has not been clarified. The aim of the present study was to assess the association between the presence of EIMs and the development of pouchitis in patients with ulcerative colitis after IPAA.

## Methods

This systematic review and meta‐analysis was conducted according to PRISMA guidelines^18^.

### Literature search and eligibility criteria

Ovid MEDLINE and Cochrane Library databases were searched systematically to identify relevant articles published to May 2017. Studies investigating preoperative risk factors for the development of pouchitis were searched for using the search terms ‘ulcerative colitis’ and ‘pouchitis’ or ‘pouch surgery’ or ‘ileal pouch anal anastomosis’ or ‘IPAA’ and ‘complication’.

The search was limited to RCTs and observational studies written in English. Studies with information on the presence of EIMs in relation to the development of pouchitis were included. Studies on familial adenomatous polyposis were excluded. The diagnostic criteria of pouchitis are essential, and it is recommended^19^, ^20^, ^21^ that the diagnosis of pouchitis is based on clinical symptoms and endoscopic confirmation of inflammation in the ileal pouch. Thus, publications in which the diagnosis was made without endoscopic features were excluded. Non‐English studies, case reports, and studies that focused only on children were also excluded.

PSC is one of the major EIMs in Western countries, and its association with pouchitis is the most frequently investigated among all EIMs. Thus, an additional attempt was made to elucidate the association of PSC and pouchitis.

### Study selection and data extraction

Two authors independently checked the titles and abstracts to exclude irrelevant studies and retrieve relevant articles. In the case of multiple publications from one institution, only studies for which the study periods did not overlap with one another were included, to avoid the duplication of cases. The reference lists of included articles were screened for additional studies.

Data extraction was performed independently by two authors, and a third author revised and finalized any disagreements. The final data were presented in Excel® (Microsoft, Redmond, Washington, USA) format for statistical analysis. The corresponding author of one article^22^ was contacted for additional data.

### Statistical analysis

EZR (Easy R)® for the R free software environment for statistical computing and graphics (https://www.R‐project.org/) was used for statistical analysis^23^. Summary statistic odds ratios (ORs) were calculated, and the risk of pouchitis was compared between patients with and without EIMs as well as between those with and without PSC. Both fixed‐effect and random‐effects models were employed, and a random‐effects model was accepted for a more conservative estimation of the effect of EIMs on the development of pouchitis. Forest plots were generated using pooled ORs from each study, and *P* < 0·050 was considered statistically significant for the pooled OR. Heterogeneity was calculated using *I* ^2^ values, and evaluated according to the method of Higgins *et al*.^24^. *P* < 0·100 was considered statistically significant for heterogeneity. To assess publication bias, funnel plots were created when ten or more studies were included, according to the recommendations of Sterne and colleagues^25^.

## Results

**Figure 1:**
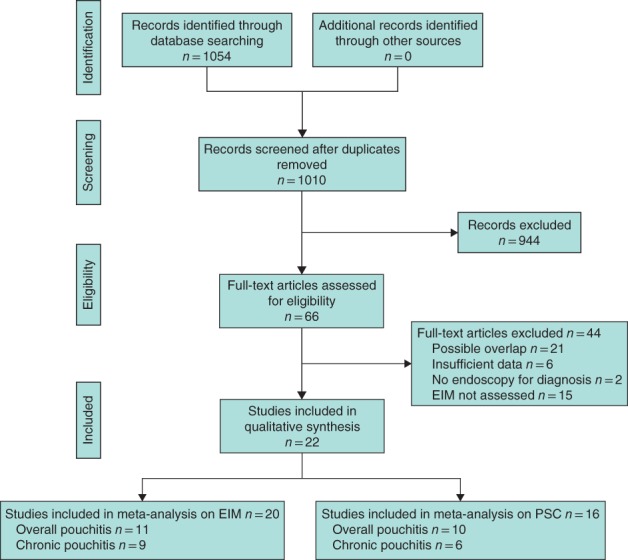
PRISMA flow diagram for the review. EIM, extraintestinal manifestation; PSC, primary sclerosing cholangitis

Of 1010 citations identified, 22 articles were included (*Fig*. 1). No relevant RCTs were retrieved. Nine and 11 observational studies were selected for the analysis of chronic pouchitis and overall pouchitis respectively in relation to EIMs (*Table S1*, supporting information)^7^, ^8^, ^12^, ^26^, ^27^, ^28^, ^29^, ^30^, ^31^, ^32^, ^33^, ^34^, ^35^, and six and ten studies respectively in relation to PSC (*Table S2*, supporting information)^13^, ^22^, ^28^, ^35^, ^36^, ^37^, ^38^, ^39^, ^40^, ^41^, ^42^. One study^42^ included both ulcerative colitis and familial polyposis. Data on patients with ulcerative colitis were extracted. Sixteen of 22 studies incorporated histology scores (5 applied the pouchitis disease activity index (PDAI)^19^), and the remaining six did not (3 applied the modified PDAI^20^). Agreement between reviewers for the assessment of study eligibility was excellent (agreement 1006 of 1010; κ statistic 0·90).

The definition of chronic pouchitis was diverse, with possible heterogeneity. EIMs included in each study varied from study to study. Most studies, however, included immune‐related EIMs such as joint disease (peripheral arthritis, ankylosing spondylitis and sacroiliitis), skin diseases (pyoderma gangrenosum and erythema nodosum), liver disease (PSC) and eye diseases (uveitis and episcleritis). In addition, some included thrombotic disorders and aphthous stomatitis. Regarding the presence of EIMs in relation to the timing of IPAA, 11 studies included exclusively preoperative EIMs, four included both preoperative and postoperative EIMs, and seven did not provide this information. The risk of bias of individual studies was assessed according to the Risk of Bias Assessment Tool for Nonrandomized Studies (RoBANS)^43^ (*Table S3*, supporting information).

### Extraintestinal manifestations and overall pouchitis

**Figure 2:**
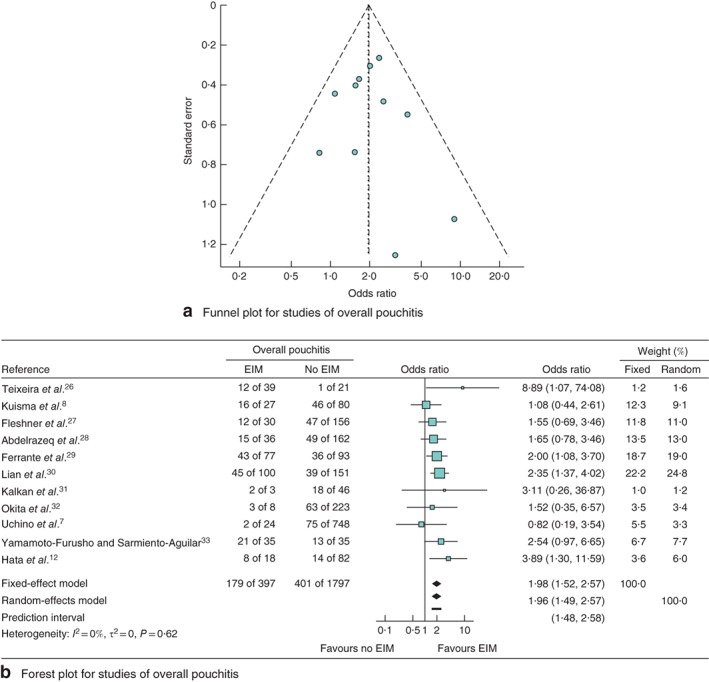
Meta‐analysis of the effect of extraintestinal manifestations (EIMs) on the development of overall pouchitis. **a** Funnel plot for studies of overall pouchitis. **b** Forest plot comparing the incidence of overall pouchitis in patients with ulcerative colitis who did or did not develop EIMs following ileal pouch–anal anastomosis. Odds ratios are shown with 95 per cent confidence intervals

The effect of EIMs on overall pouchitis was analysed using 11 papers comprising 2194 patients who received IPAA. Funnel and forest plots for overall pouchitis are shown in *Fig*. 2. The funnel plot was almost symmetrical, indicating limited publication bias.

An integrated analysis showed that the presence of EIMs was significantly associated with overall pouchitis (OR 1·96, 95 per cent c.i. 1·49 to 2·57; *P* < 0·001) with low heterogeneity (*I* ^2^ = 0 per cent, *P* = 0·62).

### Extraintestinal manifestations and chronic pouchitis

**Figure 3:**
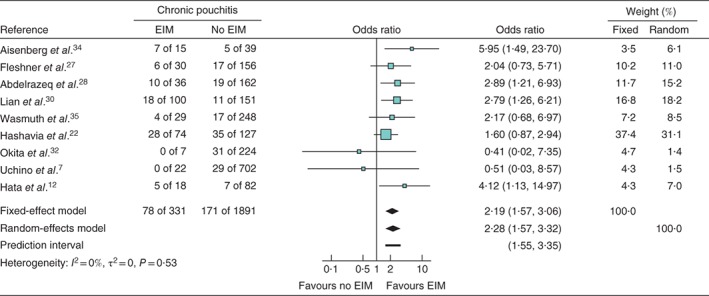
Meta‐analysis of the effect of extraintestinal manifestations (EIMs) on the development of chronic pouchitis. Forest plot comparing the incidence of chronic pouchitis in patients with ulcerative colitis who did or did not develop EIMs following ileal pouch–anal anastomosis. Odds ratios are shown with 95 per cent confidence intervals

The association of EIM with chronic pouchitis was analysed using nine papers comprising 2222 patients. The forest plot for the effect of EIMs on chronic pouchitis is shown in *Fig*. 3. An integrated analysis showed that the presence of EIMs was also significantly associated with chronic pouchitis (OR 2·28, 95 per cent c.i. 1·57 to 3·32; *P* = 0·001), with low heterogeneity (*I* ^2^ = 0 per cent, *P* = 0·53).

### Primary sclerosing cholangitis and overall pouchitis

**Figure 4:**
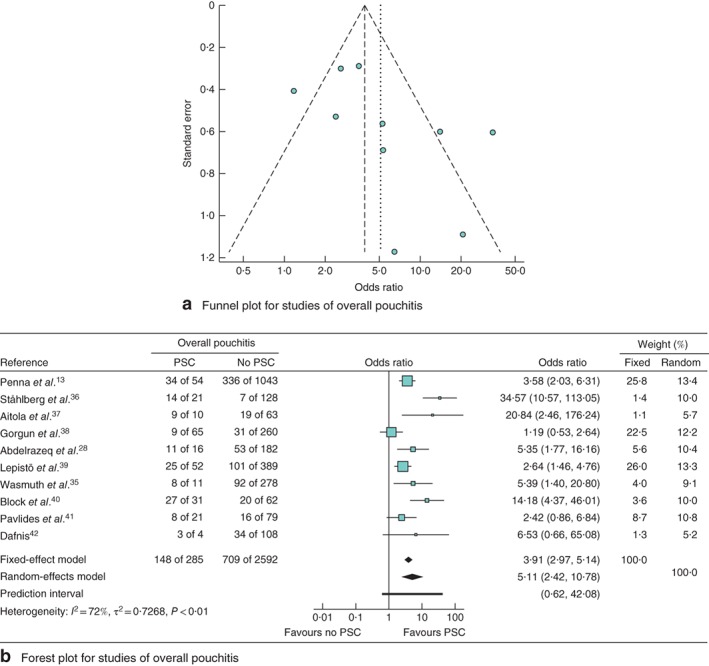
Meta‐analysis of the effect of primary sclerosing cholangitis (PSC) on the development of overall pouchitis. **a** Funnel plot for studies of overall pouchitis. **b** Forest plot comparing the incidence of overall pouchitis in patients with ulcerative colitis who did or did not develop PSC following ileal pouch–anal anastomosis. Odds ratios are shown with 95 per cent confidence intervals

Two papers provided detailed and sufficient data on PSC of the 13 articles in the analysis of the effect of EIMs on the development of overall pouchitis. Eight other articles investigating the association of PSC with pouchitis were included. These ten publications comprised 2877 patients with ulcerative colitis who underwent IPAA. Funnel and forest plots for the effect of PSC on overall pouchitis are shown in *Fig*. 4. The funnel plot for overall pouchitis was distributed asymmetrically, indicating the presence of publication bias. An integrated analysis showed that PSC was also significantly associated with overall pouchitis (OR 5·11, 95 per cent c.i. 2·42 to 10·78; *P* < 0·001), but with statistically significant heterogeneity (*I* ^2^ = 72 per cent, *P* < 0·01).

### Primary sclerosing cholangitis and chronic pouchitis

**Figure 5:**
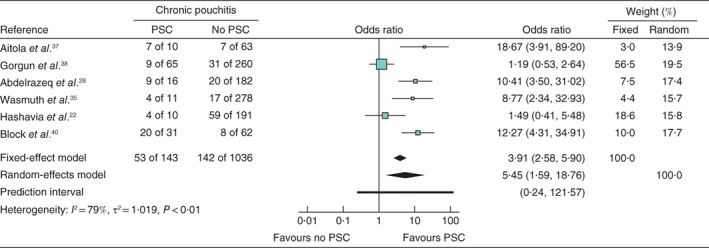
Meta‐analysis of the effect of primary sclerosing cholangitis (PSC) on the development of chronic pouchitis. Forest plot comparing the incidence of chronic pouchitis in patients with ulcerative colitis who did or did not develop PSC following ileal pouch–anal anastomosis. Odds ratios are shown with 95 per cent confidence intervals

Six papers comprising 1179 patients were included to assess the association between PSC and chronic pouchitis. The forest plot for the effect of PSC on chronic pouchitis is shown in *Fig*. 5. An integrated analysis showed that the presence of PSC was significantly associated with chronic pouchitis (OR 5·45, 95 per cent c.i. 1·59 to 18·76; *P* = 0·017), with considerable heterogeneity (*I* ^2^ = 79 per cent, *P* < 0·01).

## Discussion

Both overall and chronic pouchitis occurred significantly more often when EIMs were present. Patients with EIMs may benefit from preoperative and postoperative counselling on the need for long‐term follow‐up. Some RCTs^44^, ^45^ have demonstrated that probiotics are effective for the prevention and maintenance of pouchitis in the general population with ulcerative colitis, and it may be beneficial to prescribe probiotics for the prevention of pouchitis in patients with EIMs after IPAA.

The authors have reported the association of EIMs and development of both overall and chronic pouchitis previously^12^. The present meta‐analysis supports these findings. The authors hypothesize that patients with EIMs may have an abnormal immune response to more diverse immune targets that may cause pouchitis, as well as EIMs such as arthritis, uveitis, pyoderma gangrenosum, PSC and erythema nodosum. Pouchitis is clearly associated with an abnormal immune response, as patients with familial adenomatous polyposis undergoing IPAA develop pouchitis less frequently than those with ulcerative colitis^11^. Antineutrophil cytoplasmic antibody‐positive patients with ulcerative colitis were reported to be at risk of chronic pouchitis^16^.

PSC was also associated with the development of pouchitis. Regarding other EIMs, such as arthritis and pyoderma gangrenosum, only a few publications have investigated the association with pouchitis, so their data could not be integrated. Although PSC is one of the most frequently occurring EIMs in Western countries, the incidence of PSC is reported to be lower in Japan^46^. In three articles from Japan^7^, ^12^, ^32^, the incidence of PSC was too low to be incorporated in the pooled analysis assessing the effect of PSC on the development of pouchitis. Two Western studies^28^, ^35^ investigated the effect of PSC and EIMs on the development of pouchitis. In the present pooled analysis, the heterogeneity of included studies was significant for both overall and chronic pouchitis.

It should be stressed that diagnostic criteria for pouchitis are essential for the precise elucidation of the risk of pouchitis, because symptoms did not necessarily correlate with endoscopic and histological findings in the pouch^47^. Symptoms of pouchitis may occur in patients with irritable pouch syndromes, Crohn's disease of the pouch, or cuffitis^9^, ^21^, ^48^. Therefore, pouchoscopy is unavoidable to make a definite diagnosis of pouchitis^9^.

There were several limitations of this study. The design of all included studies was observational. Selection bias and heterogeneity is therefore inherently present. In particular, heterogeneity was statistically significant in the analysis of PSC. Several RCTs investigated the preventive effectiveness of medications such as probiotics, but data on the presence of EIMs were not detailed. The authors could not access Embase for this meta‐analysis, which might have captured some relevant publications. Most included papers used logistic rather than Cox regression in multivariable analysis. As the event of pouchitis occurred over time, it would have been better if time had been taken into account and expressed as a hazard ratio. The observation period in most studies, however, was long enough with an average of 5–10 years. The diagnosis of chronic pouchitis was not uniform among the studies. Although most papers adopted long‐term antibiotic use (more than 4 weeks) or unresponsiveness to antibiotic treatment as the definition of chronic pouchitis, the definition differed slightly from study to study.

## Supporting information


**Table S1** Characteristics of included studies for extraintestinal manifestations and pouchitis
**Table S2** Characteristics of included studies for primary sclerosing cholangitis and pouchitis
**Table S3** Risk of bias of individual studies according to the RoBANS
