# Supplementary material for: Meta‐analysis of the association of extraintestinal manifestations with the development of pouchitis in patients with ulcerative colitis
Source: BJS Open. 2019 Mar 13;3(4):436–44. doi: 10.1002/bjs5.50149 (PMC6706792; doi:10.1002/bjs5.50149)
Supplement: Supplementary file 1 — Table S1 Characteristics of included studies for extraintestinal manifestations and pouchitis Table S2 Characteristics of included studies for primary sclerosing cholangitis and pouchitis Table S3 Risk of bias of individual studies according to the RoBANS [file BJS5-3-436-s001.docx]

**BJS5_50149**

**Meta-analysis of the association of extraintestinal manifestations with the development of pouchitis in patients with ulcerative colitis**

**K. Hata, S. Okada, T. Shinagawa, T. Toshiaki, K. Kawai and H. Nozawa**

**Table S1 Characteristics of included studies for extraintestinal manifestations and pouchitis**

|  |  |  |  | **EIMs** | | **No EIMs** | |  |
| --- | --- | --- | --- | --- | --- | --- | --- | --- |
| **Study** | | **Study design** | **n** | **Pouchitis (%)** | **No pouchitis (%)** | **Pouchitis (%)** | **No pouchitis (%)** | **Association** |
| **Overall pouchitis** | |  |  |  |  |  |  |  |
| Teixeira ^26^ | | cohort | 60 | 12 (31) | 27 (69) | 1 (5) | 20 (95) | yes |
| Kuisma ^8^ | | cohort | 107 | 16 (59) | 11 (41) | 46 (58) | 34 (42) | yes in AS |
| Fleshner ^27^ | | cohort | 186 | 12 (40) | 18 (60) | 47 (30) | 109 (70) | no |
| Abdelrazeq ^28^ | | cohort | 198 | 15 (42) | 21 (58) | 49 (30) | 113 (70) | no |
| Ferrante ^29^ | | cohort | 170 | 43 (56) | 34 (44) | 36 (39) | 57 (61) | yes |
| Lian ^30^ | | cohort | 251 | 45 (45) | 55 (55) | 39 (26) | 112 (74) | Yes |
| Kalkan ^31^ | | cohort | 49 | 2 (67) | 1 (33) | 18 (39) | 28 (61) | No |
| Okita ^32^ | | cohort | 231 | 3 (38) | 5 (63) | 63 (28) | 160 (72) | No |
| Uchino ^7^ | | cohort | 772 | 2 (8) | 22 (92) | 75 (10) | 673 (90) | No |
| Yamamoto-Furusho^33^ | | cohort | 70 | 21 (60) | 14 (40) | 13 (37) | 22 (63) | No |
| Hata ^12^ | | cohort | 100 | 8 (44) | 10 (56) | 14 (17) | 68 (83) | Yes |
| **Chronic pouchitis** | |  |  |  |  |  |  |  |
| Aisenberg ^34^ | | cohort | 54 | 7 (47) | 8 (53) | 5 (13) | 34 (87) | yes |
| Fleshner ^27^ | | cohort | 186 | 6 (20) | 24 (80) | 17 (11) | 139 (89) | yes, in COX |
| Abdelrazeq ^28^ | | cohort | 198 | 10 (28) | 26 (72) | 19 (12) | 143 (88) | yes |
| Lian ^30^ | | cohort | 251 | 18 (18) | 82 (82) | 11 (7) | 140 (93) | yes |
| Wasmuth ^35^ | | cohort | 277 | 4 (14) | 25 (86) | 17 (7) | 231 (93) | no |
| Hashavia ^22^ | | cohort | 201 | 28 (38) | 46 (62) | 35 (28) | 92 (72) | no |
| Okita ^32^ | | cohort | 231 | 0 (0) | 7 (100) | 31 (14) | 193 (86) | no |
| Uchino ^7^ | | cohort | 724 | 0 (0) | 22 (100) | 29 (4) | 673 (96) | no |
| Hata ^12^ | | cohort | 100 | 5 (28) | 13 (67) | 7 (9) | 75 (91) | yes |

AS; ankylosing spondylitis, EIM; extra-intestinal manifestation

**Table S2 Characteristics of included studies for primary sclerosing cholangitis and pouchitis**

|  |  |  | **PSC** | | **No PSC** | |  |
| --- | --- | --- | --- | --- | --- | --- | --- |
| **Study** | **Study design** | **n** | **Pouchitis (%)** | **No pouchitis (%)** | **Pouchitis (%)** | **No pouchitis (%)** | **Association** |
| **Overall pouchitis** |  |  |  |  |  |  |  |
| Penna ^13^ | cohort | 1097 | 34 (63) | 20 (37) | 336 (32) | 707 (68) | yes |
| Ståhlberg ^36^ | cohort | 149 | 14 (67) | 7 (33) | 7 (5) | 121 (95) | yes |
| Aitola ^37^ | cohort | 73 | 9 (90) | 1 (10) | 19 (30) | 44 (70) | yes |
| Gorgun ^38^ | case-control | 325 | 9 (14) | 56 (86) | 31 (12) | 229 (88) | no |
| Abdelrazeq ^28^ | cohort | 198 | 11 (69) | 5 (31) | 53 (29) | 129 (71) | yes |
| Lepisto ^39^ | cohort | 441 | 25 (48) | 27 (52) | 101 (26) | 288 (74) | yes |
| Wasmuth ^35^ | cohort | 289 | 8 (73) | 3 (27) | 92 (33) | 186 (67) | yes |
| Block ^40^ | case-control | 93 | 27 (87) | 4 (13) | 20 (32) | 42 (68) | yes |
| Pavlides ^41^ | case-control | 100 | 8 (38) | 13 (62) | 16 (20) | 63 (80) | no |
| Dafnis ^42^ | cohort | 112 | 3 (75) | 1 (25) | 34 (31) | 74 (69) | yes, in COX |
| **Chronic pouchitis** |  |  |  |  |  |  |  |
| Aitola ^37^ | cohort | 73 | 7 (70) | 3 (30) | 7 (11) | 56 (89) | yes |
| Gorgun ^38^ | case-control | 325 | 9 (14) | 56 (86) | 31 (12) | 229 (88) | no |
| Abdelrazeq ^28^ | cohort | 198 | 9 (56) | 7 (44) | 20 (11) | 162 (89) | yes |
| Wasmuth ^35^ | cohort | 289 | 4 (36) | 7 (64) | 17 (6) | 261 (94) | yes |
| Hashavia ^22^ | cohort | 201 | 4 (40) | 6 (60) | 59 (31) | 132 (69) | no |
| Block ^40^ | case-control | 93 | 20 (65) | 11 (35) | 8 (13) | 54 (87) | yes |

PSC; primary sclerosing cholangitis

**Table S3 Risk of bias of individual studies according to the RoBANS**

| **Study** | **Year** | Selection of participants | Confounding variables | Measurement of exposure (EIM) | Blinding of outcome assessments | Incomplete outcome data | Selective outcome reporting |
| --- | --- | --- | --- | --- | --- | --- | --- |
| Teixeira^26^ | 1999 | low | unclear | low | unclear | unclear | low |
| Gorgun^38^ | 2005 | low | low | low | unclear | unclear | high |
| Pavlides^41^ | 2014 | low | unclear | low | low | unclear | low |
| Wasmuth^35^ | 2010 | low | unclear | low | unclear | unclear | low |
| Abdelrazeq^28^ | 2008 | unclear | unclear | unclear | low | unclear | low |
| Dafnis^42^ | 2016 | low | unclear | low | unclear | unclear | low |
| Kalkan^31^ | 2012 | low | unclear | unclear | unclear | unclear | low |
| Okita^32^ | 2013 | low | unclear | low | low | unclear | low |
| Penna^13^ | 1996 | low | low | low | unclear | unclear | low |
| Uchino^7^ | 2013 | low | unclear | low | low | unclear | low |
| Aitola^37^ | 1998 | unclear | unclear | low | unclear | unclear | low |
| Aisenberg^34^ | 1995 | low | low | low | unclear | unclear | low |
| Block^40^ | 2013 | unclear | unclear | low | low | unclear | low |
| Ferrante^29^ | 2008 | unclear | unclear | low | unclear | unclear | low |
| Fleshner^27^ | 2007 | low | unclear | low | unclear | unclear | low |
| Hashavia^22^ | 2012 | low | unclear | unclear | low | unclear | low |
| Hata^12^ | 2017 | low | unclear | low | low | unclear | low |
| Kuisma^8^ | 2004 | unclear | unclear | low | low | unclear | low |
| Lian^30^ | 2009 | low | unclear | low | low | unclear | low |
| Lohmuller^11^ | 1990 | unclear | low |  | High | unclear | low |
| Ståhlberg ^36^ | 1996 | low | unclear | low | unclear | unclear | low |
| Lepisto^39^ | 2008 | low | unclear | low | unclear | unclear | low |
| Yamamoto-Furusho^33^ | 2015 | high | unclear | unclear | unclear | unclear | low |
